# Petrography and physical-mechanical evaluation of mafic-ultramafic rocks from Atud-Um Khasila, Egypt for dimension stone

**DOI:** 10.1038/s41598-026-44938-y

**Published:** 2026-04-11

**Authors:** Ahmed M. Abdel-Rahman, Mahmoud L. Abdel Latif, Mohamed Zaki Khedr, Sarah A. Mohsen, Mohamed H. Ghoneim

**Affiliations:** 1 Geology Department, Faculty of Science, Al-Azhar University, Cairo, 11884 Egypt; 2 Housing and Building National Research Center, Cairo, 11511 Egypt; 3 Department of Geology, College of Science, Taibah University, Madinah, Saudi Arabia; 4 Geology Department, Faculty of Science, Kafrelsheikh University, Kafr El-Shaikh, Egypt; 5 Geology Department, Faculty of Science, Alexandria University, Alexandria, Egypt; 6 Ashgill Australia Pty. Ltd Level, 9 - 444 Collins Street, Melbourne, VIC 3000 Australia

**Keywords:** Dimension stone, Gabbro, Serpentinite, Mafic, Physical and mechanical properties, Egypt, Materials science, Solid Earth sciences

## Abstract

This study investigates the petrography, physical, and mechanical properties of mafic-ultramafic rocks from the Atud–Um Khasila region in Egypt’s Central Eastern Desert for dimension-stone applications. Eighteen representative samples of metagabbro, olivine gabbro, and serpentinite were analyzed using integrated petrographic and geotechnical methods. Standard ASTM and EN procedures were utilized to determine bulk density, water absorption, apparent porosity, and uniaxial compressive strength (UCS). Additionally, serpentinite samples were evaluated for durability against salt crystallization and thermal shock. Petrographic analysis reveals that gabbroic rocks (metagabbro and olivine gabbro) comprise plagioclase, amphibole, pyroxene, and olivine, forming interlocking textures that may contribute to their strength. Metagabbro demonstrated high strength (74.6 MPa), low porosity (0.24%), and minimal water absorption (0.08%). Olivine gabbro also performed well, with a strength (76.8 MPa), a porosity (0.29%), and water absorption (0.11%). In contrast, serpentinites, mostly made up of antigorite, talc, and carbonate veins, showed lower strength (67.3 MPa) due to alteration but had weight losses below 1% during testing. Overall, gabbroic rocks are appropriate for dimension stone applications, while serpentinite does not meet ASTM standards. This study demonstrates that the mineralogy and texture strongly influence rock performance, emphasizing the importance of combined petrographic and geotechnical assessments in evaluating stone resources.

## Introduction

Natural stones have been essential construction materials since prehistoric times, valued for their mechanical strength, resilience, and aesthetic beauty. Natural rock resources that have been mined into slabs or blocks of particular sizes and forms are referred to as dimension stone, encompassing lithologies such as granite, limestone, marble, sandstone, basalt, and slate^1^. Their physical and ornamental qualities have supported their extensive use, ranging from polished interior slabs to structural elements in monumental architecture, such as the temples and tombs of ancient Egypt^2,3^.

Since natural stones are a vital geological resource, their demand has grown continuously worldwide, making them one of the most expensive mineral commodities globally^4^. The EN 12,670 (2019)^5^ standard defines natural building stones as materials suitable for both new construction and the conservation of heritage buildings. Technological advancements in quarrying and processing have reinforced their role as valuable geological resources, making them among the highest-value mineral commodities worldwide^4^. Critical factors influencing their selection include durability, porosity, water absorption, color, accessibility, and ease of quarrying, which are strongly dependent on the mineralogical composition, texture, weathering state, and tectonic history^6,7^.

**Fig. 1:**
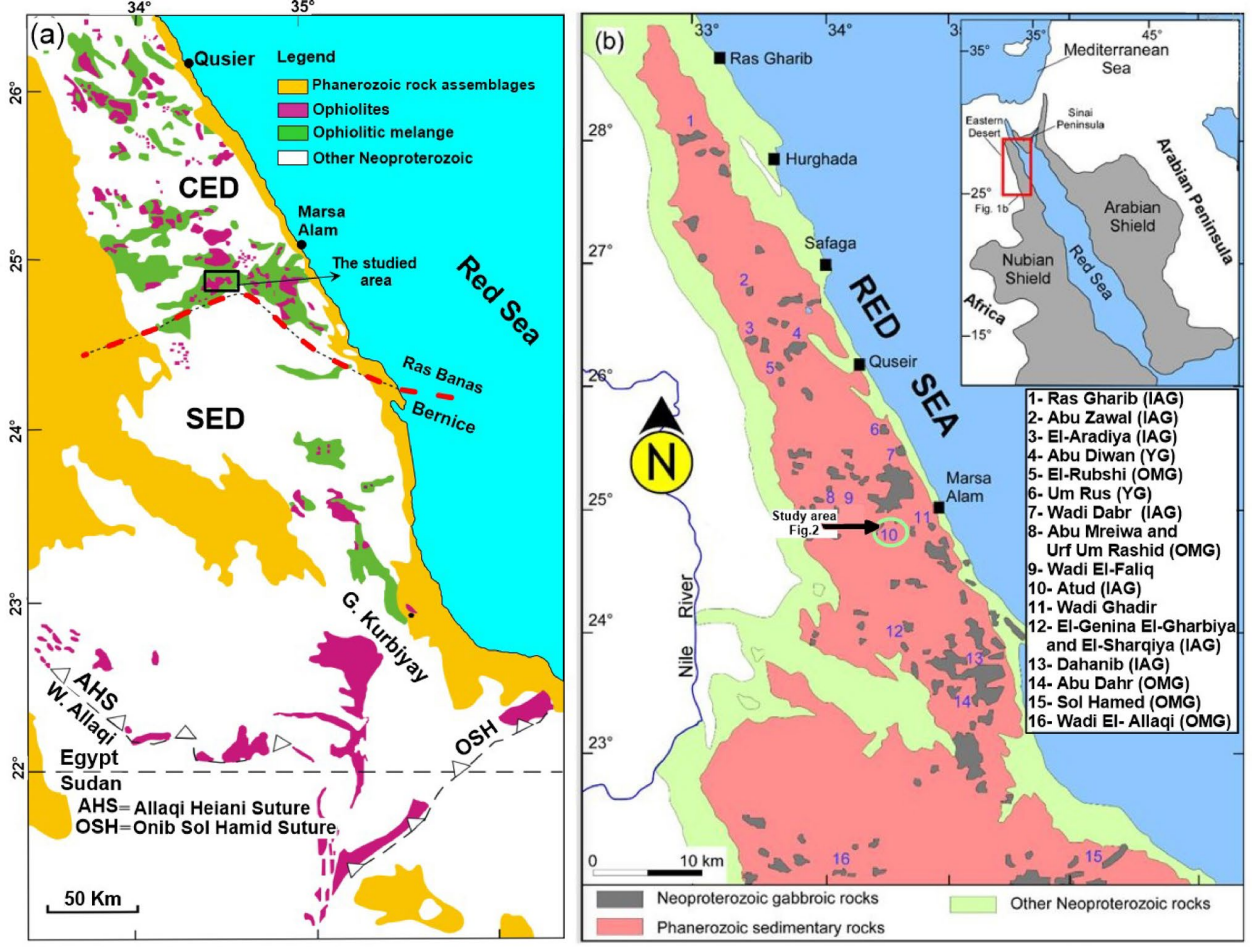
(**a**) Regional geological map showing the distribution of ophiolites in the Eastern Desert of Egypt (compiled from published literature^15^; (**b**) Distribution of various Neoproterozoic gabbroic rocks in the Eastern Desert of Egypt (modified after Abdelfadil et al. 2022^21^. *OMG*: ophiolitic metagabbro; *IAG*: island arc gabbro; *YG*: younger gabbro.

In Egypt, the Arabian-Nubian Shield (ANS) represents a geologically diverse basement terrain that hosts abundant mafic and ultramafic rocks, particularly in the central and southern regions of the Eastern Desert^8–10^. These rocks are commonly associated with dismembered ophiolite complexes, reflecting the tectono-magmatic evolution of the region^11–17^. Within these complexes, peridotites often undergo hydration, where interaction with H₂O and CO₂ transforms them into serpentinite and ophiocarbonate, respectively. Such mineralogical alterations impart distinctive physical and chemical properties that strongly influence their performance when employed as commercial construction stones^9^. Serpentinites are widely employed in tiling and ornamental applications; however, their durability is highly dependent on factors such as geochemistry, mineralogy, and mechanical properties, which vary according to their geological setting^18^(e.g., Wadi Attallah, Wadi Sodmien, Wadi Allaqi). Therefore, a comprehensive evaluation of these parameters is essential to mitigate deterioration risks and to ensure the sustainable utilization of serpentinites in building and decorative industries. The protoliths of serpentinites are predominantly highly depleted harzburgites to dunites that have experienced extensive serpentinization, facilitated by hydration and fluid-rock interaction in supra-subduction zone (SSZ) settings, particularly in fore-arc mantle wedges (Fig. 1a^15,16,19,20^;.

Neoproterozoic gabbroic rocks are widely distributed across the Egyptian Eastern Desert (Fig. 1b^21–23^;. These units typically intrude the older island-arc tonalite-trondhjemite-granodiorite (TTG) suites and are, in turn, cross-cut by younger post-collisional granitoids of Ediacaran age (~ 620 − 590 Ma^24,25^;. The emplacement of these later granites occurred during the final stages of arc amalgamation^26,27^ within the Arabian-Nubian Shield. In contrast, Phanerozoic gabbros are not isolated but form components of alkaline ring complexes, which are predominantly located in the southern parts of the Eastern Desert.

Mafic and ultramafic rocks (e.g., basalt, gabbro, diabase, and peridotite) are valued for their mechanical strength, durability, and economic accessibility. Their resistance to weathering processes such as freeze-thaw cycles, acidic rain, and salt exposure enhances their suitability for long-term structural and decorative applications (e.g., curbstones, pavements, and monuments). Economically, their global abundance and local availability reduce material and transportation costs, supporting cost-effective use. Nonetheless, sustainable quarrying and utilization practices are crucial for maintaining environmental and economic balance over time.

This study aims to evaluate the physical and mechanical properties of mafic and ultramafic rocks exposed in the Umm Khasila area, with a focus on their potential application as durable and sustainable dimension stones in the engineering and construction sectors.

## Geologic setting

The Atud-Um Khasila region, located about 60 km west of Marsa Alam on the Red Sea coast, occupies an area of approximately 205 km² in Egypt’s Eastern Desert. Structurally, the region is characterized by thrust faults, normal faults, folds, and strike-slip faults^28,29^. The dominant structural fabrics are NW-SE to NNW-SSE and NE-SW to NNE-SSW.

**Fig. 2:**
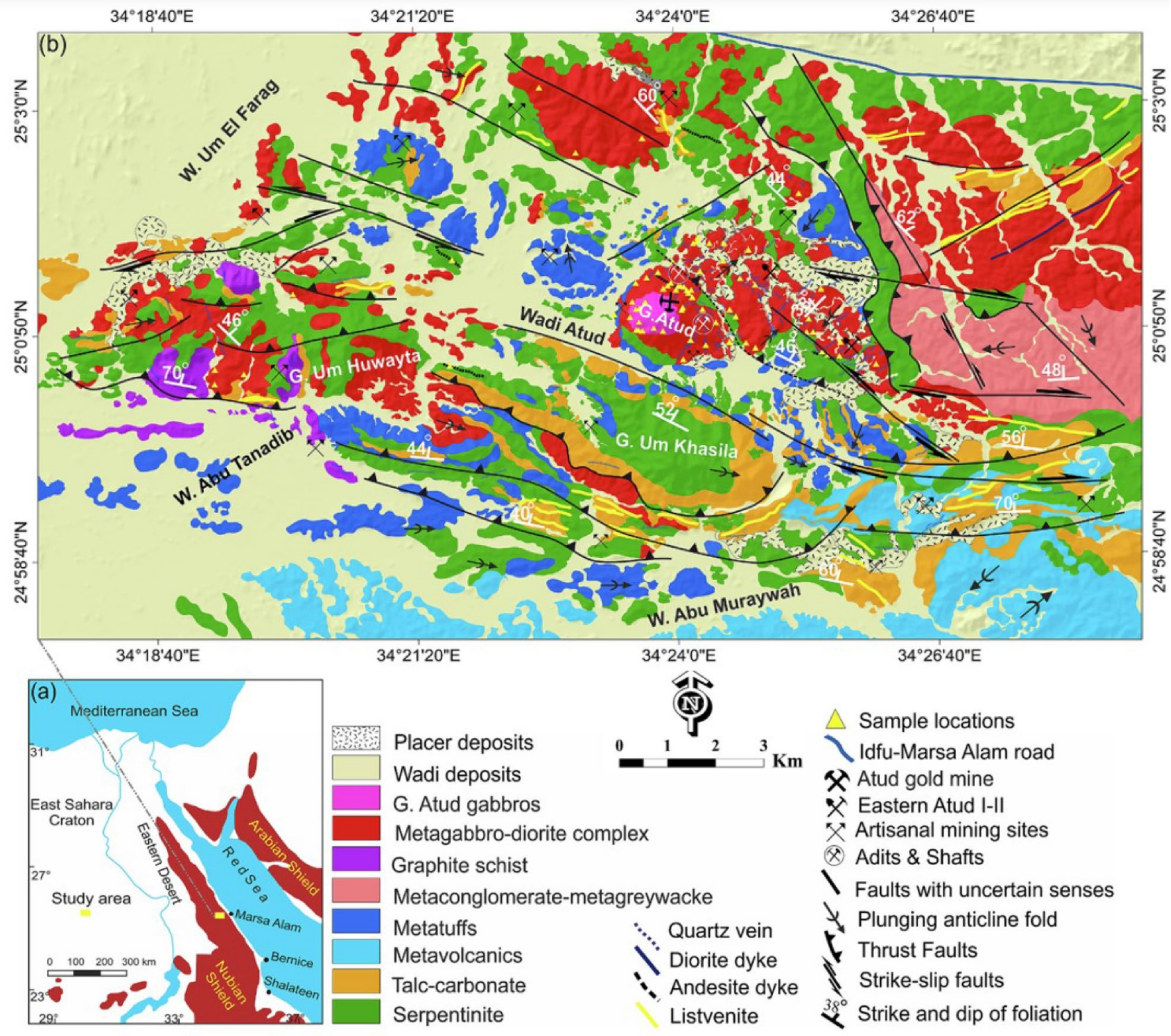
**(a**) Location of the study area, (**b**) Geological map of the Atud-Um Khasila area in the Eastern Desert of Egypt^9^.

Khedr et al. (2024b)^10^ state that the Neoproterozoic basement complex can be divided into three main rock groups: (1) ophiolitic mélange units, (2) island-arc assemblages, and (3) post-collisional mafic intrusions (Figs. 1b and 2). The distorted Cryogenian-Tonian ophiolites, together with related island-arc metavolcanics and metasediments, were intruded by the Atud gabbroic rocks^23^. The metagabbro-diorite complex and the G. Atud fresh gabbros (formerly known as younger gabbros) make up the mafic plutonic assemblage (Figs. 1b and 2).

**Fig. 3:**
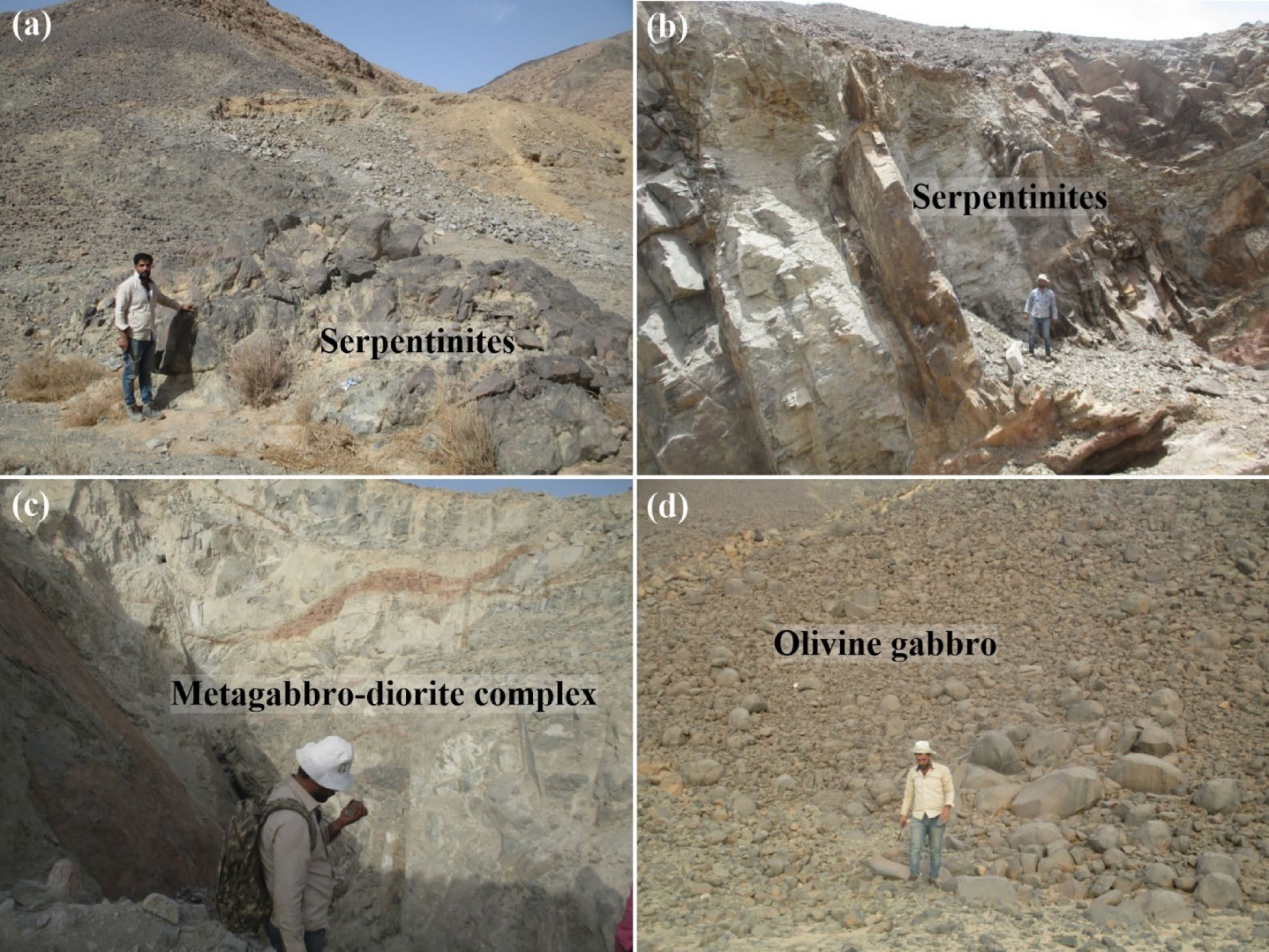
Field photographs of the mafic and ultramafic rocks at Atud-Um Khasila; **(a**,** b)** low hills of serpentinite. **(c)** Metasomatic alteration zones of metagabbro-diorite complex. **(d)** Onion-shaped olivine gabbros.

The large serpentinite outcrops south of Gabal Atud exhibit variable deformation and foliation, with metagabbro-diorite intrusions intruding into serpentinites, metatuffs, and metaconglomerate-metagreywack (Fig. 3a, b). We noted intrusive contacts between gabbroic rocks and surrounding units. In the central area west of G. Um Huwaytat, metagabbro-diorite rocks intrude into serpentinites, metatuffs, and metaconglomerate-metagreywack (Fig. 3c).

However, the olivine gabbro has encroached on the old metagabbro-diorite complex, exhibiting metasomatic alteration zones and intrusive contacts (Fig. 3d). They occur at the top and middle of the G. Atud and have a bulbous form, resembling fresh younger gabbros (Fig. 2b). The metasomatic alteration zones are common along NW-SE direction (especially metagabbro) and surrounding shear zones in the present study.

## Materials and methods

A total of 18 representative rock samples were collected from the study area, comprising metagabbro (4 samples), olivine gabbro (3 samples), and serpentinite (11 samples), which represent the main mafic-ultramafic lithologies in the study area and were evaluated for their potential suitability for building and dimension stone.

### Mineralogical and petrographic analyses

It is crucial to understand the mineralogical and petrographic qualities of rocks to attribute their characteristics accurately. The current study utilized thin-section images obtained from a camera mounted on a polarizing microscope to calculate the percentages of mineralogical and petrographic attributes. Point counting was performed following the standard petrographic approaches described in references^30–34^.

### Geotechnical tests

#### Physical and mechanical properties

Geotechnical investigations were conducted to analyze the behavior of rocks by testing their physical and mechanical properties. These properties were measured at the Housing and Building National Research Center (HBRC) in the Laboratory of Raw Building Materials and Processing Technology Research Institute, Dokki, Egypt. All physico-mechanical experiments were conducted on the studied rock types in accordance with ASTM standards. At the same time, additional tests, such as salt crystallization (EN 12370:2020)^35^ and thermal shock (EN 14066:2013)^36^, were performed exclusively on the serpentinite samples, as these rocks are generally more vulnerable to weathering processes due to their mineralogical composition and alteration features, compared to the more mechanically competent gabbroic rock.

**Fig. 4:**
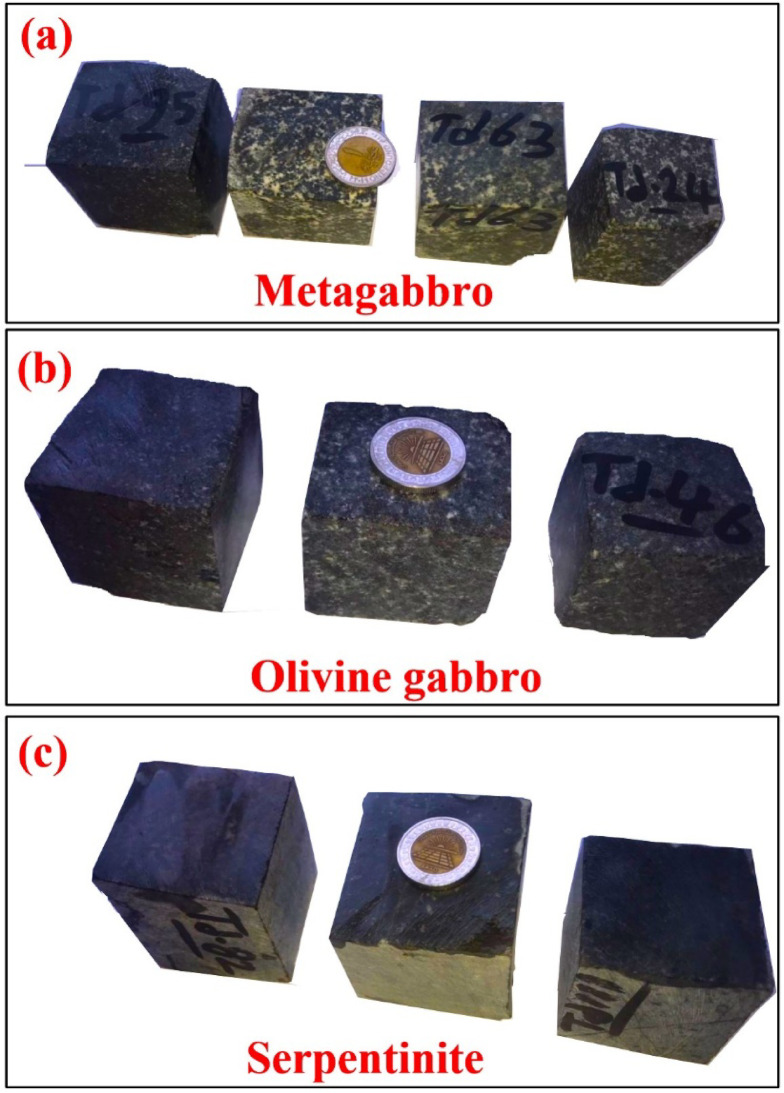
Hand specimen cubic of the mafic-ultramafic types at Atud area; (**a**) Metagabbro, (**b**) Olivine gabbro, (**c**) Serpentinite.

Representative blocks were cut into regular cubes of 50 × 50 × 50 mm using a cutting machine (Fig. 4). Thirteen specimens metagabbro (4 samples), olivine gabbro (3 samples), and serpentinite (6 samples) were tested for physical and mechanical properties, including bulk density, water absorption, and apparent porosity, in accordance with ASTM C20^37^ and ASTM C97^38^ standards. The mechanical performance was evaluated through uniaxial compressive strength (UCS) testing, following the procedures outlined in ASTM C170^39^, ASTM C99^40^, and ASTM C880^41^. The following relationships were applied in the calculations: bulk density (g/cm³) = weight (M)/volume (V); water absorption (%) = (final weight – initial weight)/initial weight × 100; apparent porosity (%) = bulk density × water absorption; and compressive strength (MPa) = load (W)/cross-sectional area (A).

#### Salt crystallization resistance

The salt crystallization resistance of five serpentinite samples was tested in accordance with EN 12370:2020^35^. Cubic specimens (40 ± 1 mm) were cut, ground, oven-dried at 105 ± 5 °C to constant mass (Md), cooled, and reweighed (Md₁). Each test cycle involved 2–4 h of immersion in a 14% Na₂SO₄·10 H₂O solution, followed by 18 h of drying at 105 °C. The specimen mass (Mdn) was recorded after each cycle. After 15 cycles, the samples were immersed in water (23 ± 5 °C) for 24 ± 1 h, cleaned, dried to a constant mass, and weighed (Mf). The relative mass change (ΔM), indicating salt crystallization damage, was calculated as:

1 $$\:\varDelta\:M=\frac{Mf-Md1\:}{Md}\times\:100$$

#### Thermal shock (Heating-Cooling cycles)

Thermal shock in rocks occurs due to rapid heating or cooling, producing internal temperature gradients and thermal stresses that can exceed the rock’s strength^42–44^. This leads to the formation of microcracks, increased fracture connectivity, and alterations in properties, including bulk density, compressive strength, and tensile strength^45,46^.

In this study, the thermal shock resistance of five serpentinite samples was tested in accordance with BS EN 14066:2013^36^ by subjecting the samples to 50 cycles of drying at 105 ± 5 °C and immersion in distilled water at 20 ± 5 °C. The mass change (ΔM), indicating thermal damage, was calculated as:

2 $$\:{\Delta\:}M=\frac{{M}_{n}-{M}_{0}}{{M}_{0}}\times\:100$$

Where, ΔM: represents the mass loss or gain %, M_0_: the original dry mass g, and Mn: dry mass of the sample g after 50 cycles.

## Results and discussion

### Petrographic characterization of the studies rocks

**Fig. 5:**
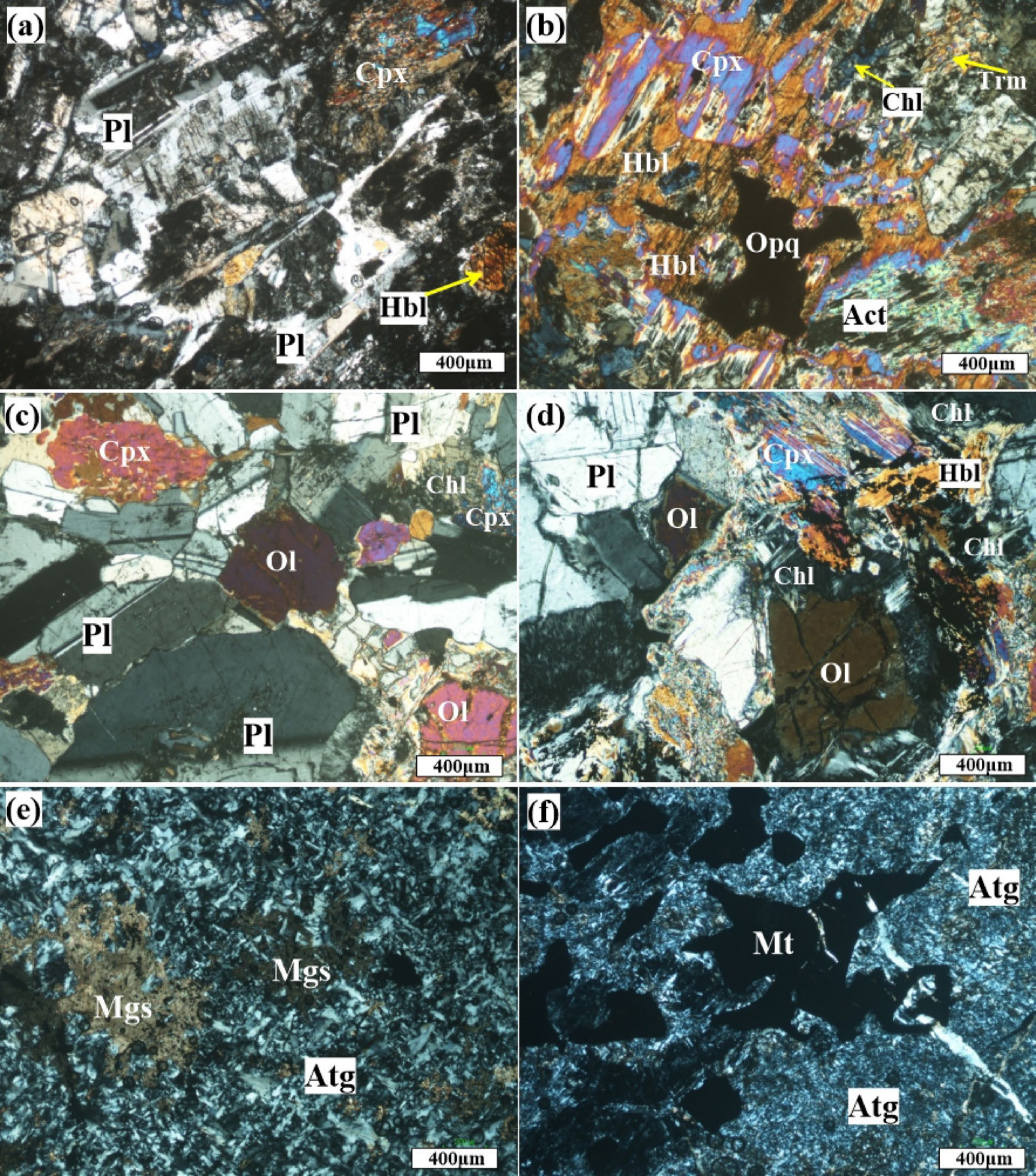
Photomicrographs showing textures and mineral assemblages of metagabbro (**a**,** b**), olivine gabbro (**c**,** d**), and serpentinites (**e**,** f**); (**a**) coarse deformed grains of plagioclase (Pl) crystals altered to cloudy kaolinite, surrounded by clinopyroxene (Cpx) and hornblende. (**b**) clinopyroxene (Cpx) altered to hornblende (Hbl), tremolite (Trm), chlorite (Chl). (**c**) olivine (Ol) crystals hosted in coarse plagioclase (Pl). (**d**) plagioclase (Pl) altered to saussurite associated with clinopyroxene (Cpx) altered to hornblende (Hbl) and chlorite with little opaques. (**e**) mesh texture of antigorite (Atg), (**f**) patches of magnetite (Mt) crystals hosted in antigorite (Atg). Abbreviations: *Pl* plagioclase, *Cpx* clinopyroxene, *Hbl* hornblende, *Chl* chlorite, *Trm* tremolite, *Act* Actinolite, *Opq* opaque minerals, Ol Olivine, *Mgs* magnesite, *Atg* antigorite, *Mt* magnetite.

As illustrated in Fig. 5, the metagabbro is composed essentially of plagioclase (60–70%) and amphibole (25–30%) with rare pyroxene. Quartz (< 5%) and opaque minerals (5–8%) are the main accessories. Epidote, chlorite, sericite, kaolinite, and carbonates are the main secondary minerals. These rocks are characterized by hypidiomorphic, diabasic, and ophitic to subophitic textures. Plagioclase occurs in two distinct generations. The primary type, representing less than 5% of the total, consists of relatively fresh, subhedral to euhedral crystals (Fig. 5a). The secondary type, which is more abundant (~ 95%), appears as fine, altered crystals filling interstitial spaces (Fig. 5b). Plagioclase crystals are altered to sericite, kaolinite, and chlorite. Amphiboles are represented by hornblende, actinolite, and tremolite. Hornblende occurs as medium- to coarse-grained, high-relief, idiomorphic to subidiomorphic, prismatic crystals, and sometimes alters to chlorite (Fig. 5b). Tremolite-actinolite occurs as idiomorphic to xenomorphic crystals, of pale brown color, showing weak pleochroism. It occurs as alteration products of pyroxene (Fig. 5b).

Olivine gabbro is composed of amphibole (40–45%), plagioclase (30–40%), clinopyroxene (20–25%), olivine (5–20%), and with minor amounts of chlorite, sericite, talc, and carbonate. Chlorite and sericite occur as alteration products of plagioclase and pyroxene, respectively. The olivine gabbro is distinguished by corona and ophitic to subophitic textures. Plagioclase (An_82–88_) is of calcic composition (anorthite to labradorite). It is represented by euhedral and subhedral crystals (Fig. 5c). Clinopyroxene crystals are characterized by high interference colors. They are partly to totally altered to hornblende, chlorite, and tremolite-actinolite (Figs. 5c, d). Olivine occurs as granular crystals up to 1.5 mm in size. Hornblende occurs as medium-grained, euhedral to subhedral prismatic crystals, measuring 2.5 to 3 mm in size. It exhibits high relief and is brown in color, pleochroic from yellowish green to brown (Fig. 5c, d).

Serpentinites comprise serpentine minerals (60–80%), a minor amount of carbonates (5–25%), and opaque minerals (5–10%). The serpentine minerals are represented mainly by antigorite as the main constituent. Antigorite is flaky, colorless, xenomorphic with feather-shaped aggregates. Mesh texture is dominant. Carbonates exhibit interstitial patches as an alteration product of serpentine minerals (Fig. 5e). They occur as irregular, intersecting veinlets and fine-grained aggregates (Fig. 5e, f). Opaques are mainly composed of magnetite and Cr-spinel crystals and occur as coarse patches that fill the cracks and fractures (Fig. 5f).

### Physico-mechanical properties of the studied mafic-ultramafic rocks

**Fig. 6:**
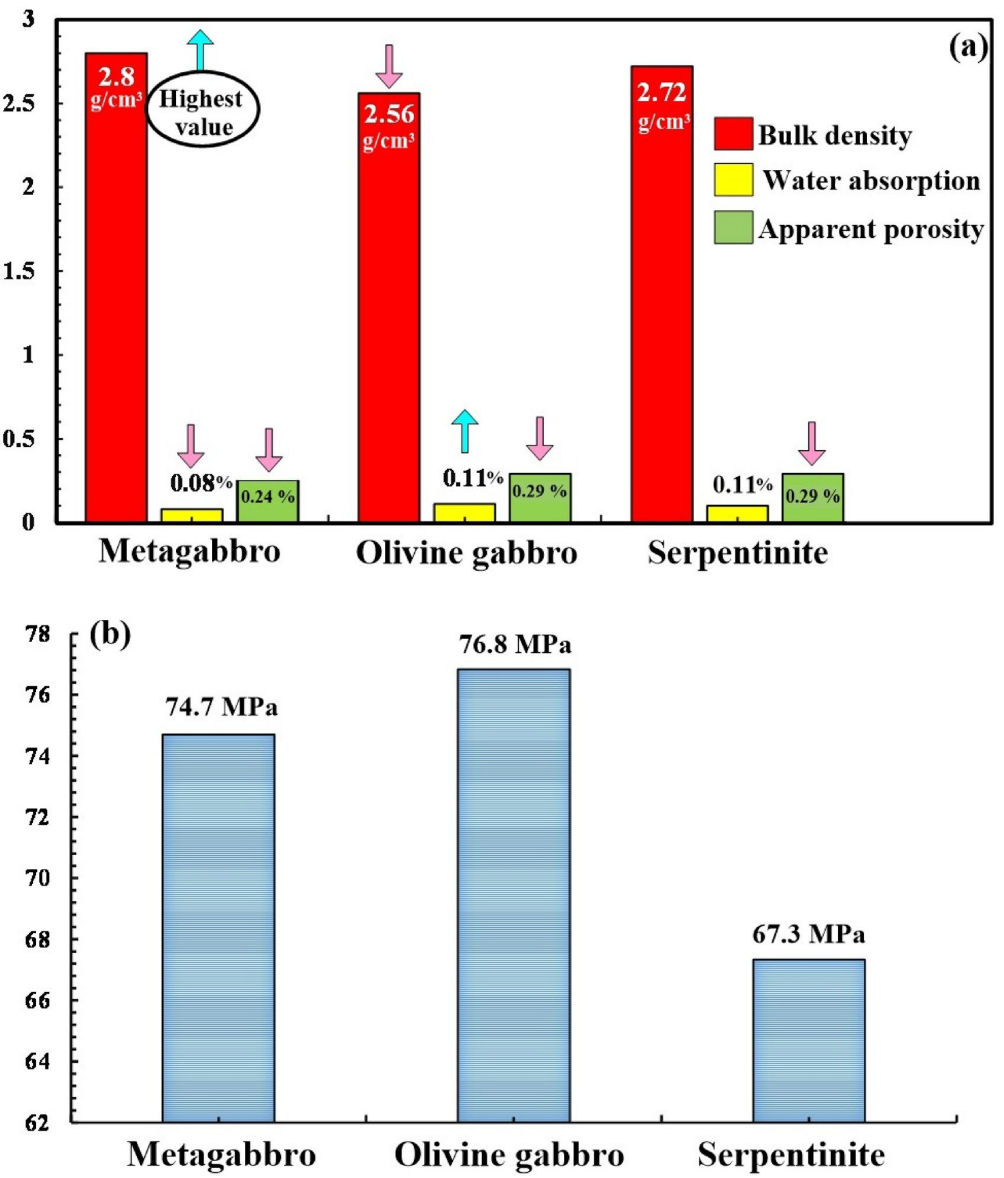
Histogram showing (**a**) the comparison between the average of the different physical properties (bulk density (g/cm³), water absorption (%), apparent porosity (%)) in the studied samples. (**b**) average values of the compressive strength (MPa) of the present samples.

Table 1 presents the main results and statistical parameters derived from the physical and mechanical testing of 13 mafic-ultramafic rock samples. Figure 6a shows that bulk density ranges from 2.70 to 2.90 g/cm³ in metagabbro (average 2.80 g/cm³), 2.32–2.70 g/cm³ in olivine gabbro (average 2.56 g/cm³), and 2.50–3.00 g/cm³ in serpentinite (average 2.72 g/cm³). Similarly, the lowest values of water absorption (0.08%) and apparent porosity (0.24%) are also recorded in metagabbro, with higher values observed in olivine gabbro and serpentinite (Fig. 6a). Uniaxial compressive strength (UCS) ranges from 73.5 to 78.5 MPa in olivine gabbro (average 76.8 MPa) and from 72.5 to 76 MPa in metagabbro (average 74.6 MPa). In contrast, serpentinite shows lower UCS values, ranging from 63.5 to 72 MPa (average 67.3 MPa), and the highest standard deviation (3.2 MPa) from the dataset. (Fig. 6b).

**Table 1:** Physical and mechanical values of the examined gabbroic (metagabbro and olivine gabbro) and serpentinite rocks.

Sample code	Bulk density (g/cm^3^)	Water absorption (%)	Apparent porosity (%)	Uniaxial Compressive strength (MPa)
**Metagabbro**				
TD24	2.70	0.16	0.40	72.5
TD63	2.90	0.02	0.05	76
TD74	2.90	0.10	0.39	75
TD95	2.70	0.05	0.13	75
Average	2.80	0.08	0.24	74.6
*St.Dev*	0.11	0.06	0.17	1.5
**Olivine gabbro**				
TD3	2.32	0.08	0.18	73.5
TD46	2.67	0.14	0.37	78.5
TD139	2.70	0.12	0.32	78.5
Average	2.56	0.11	0.29	76.8
St.Dev	0.21	0.03	0.09	2.9
**Serpentinite**				
Td91	2.50	0.06	0.15	63.5
Td92	3.00	0.10	0.30	72
Td82	2.97	0.27	0.80	70
Td 111	2.60	0.08	0.23	64.5
Td 117	2.60	0.04	0.10	66.5
Td 118	2.70	0.07	0.19	67.5
Average	2.72	0.10	0.29	67.3
*St.Dev*	0.20	0.08	0.25	3.2

**Fig. 7:**
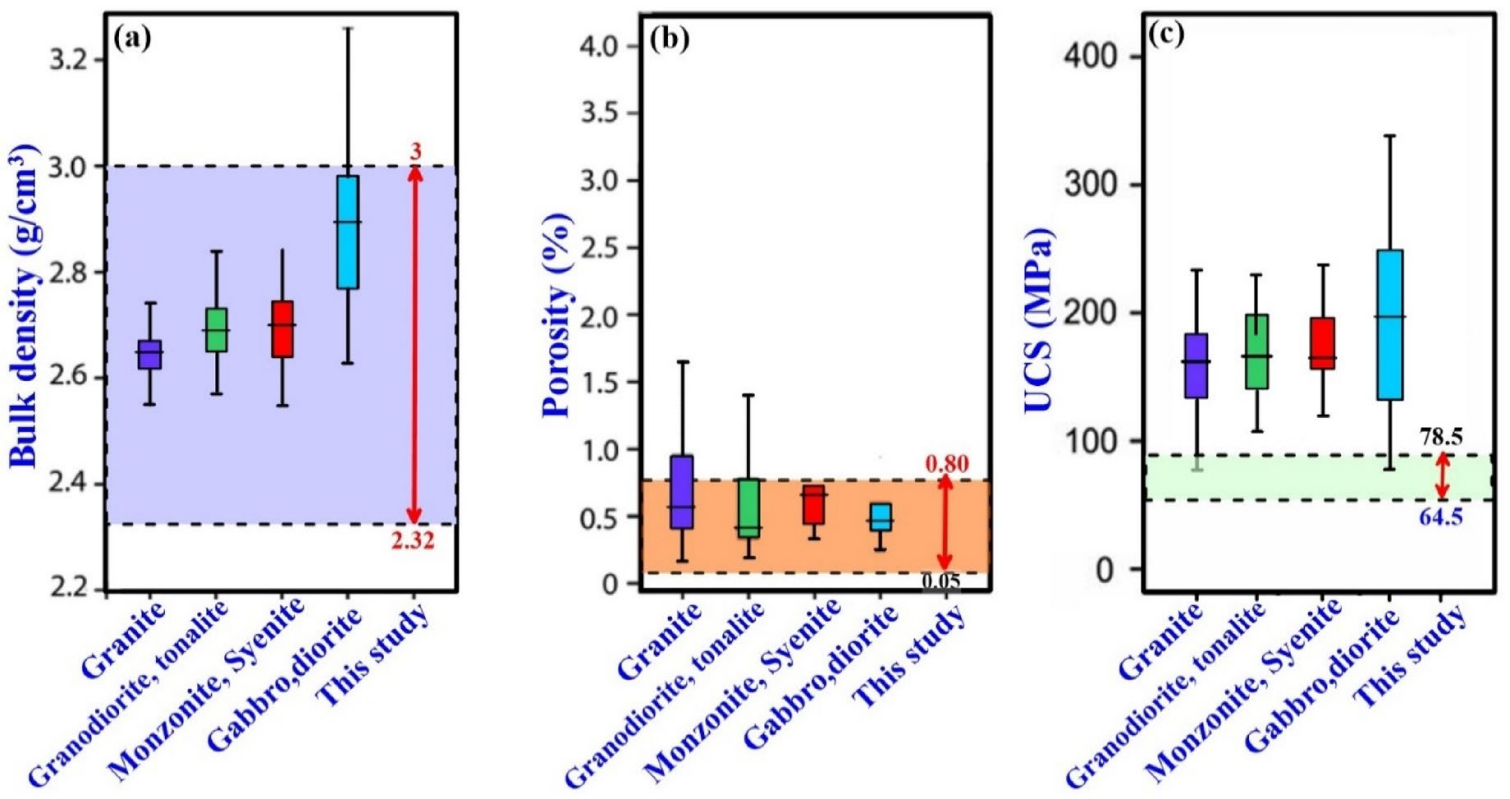
The average of the present study (each red arrow color) compared with the global average of (**a**) bulk density (g/cm³), (**b**) porosity (%), (**c**) uniaxial compressive strength values UCS (MPa), after^47^.

According to Mosch (2009)^47^, the typical ranges of bulk density, porosity, and uniaxial compressive strength in plutonic rocks are 2.62–2.90 g/cm³, 0.3–0.9%, and 131–250 MPa, respectively (Fig. 7). The measured values fall within the acceptable range for bulk density and apparent porosity but fail to meet the uniaxial compressive strength standards outlined by Mosch (2009)^47^. Based on Carmichael’s (1989)^48^ classification, the tested rocks can be categorized as medium-strength lithologies, with uniaxial compressive strength values ranging from 55 to 110 MPa. Under ASTM C170 (1999)^39^ specifications, the serpentinite samples from the Um Khasila area exhibit generally low uniaxial compressive strength (UCS) values, falling below the requirements of ASTM C1526 (2008)^49^ for dimension stones, except for two samples (Td82 and Td92) that approach the minimum threshold. This behavior indicates that most serpentinite samples from the study area have limited suitability for dimension-stone applications based on mechanical criteria.

The relatively weak performance of serpentinites can be attributed to deformation and shearing processes, which promote the development of calcite-filled veins that weather more rapidly than the host rock^50^, as well as the effects of alteration and structure on rock behavior^51^. In contrast, the serpentinite samples from Umm Khasila exhibit lower apparent porosity and water absorption than serpentinites from other Egyptian localities, such as Wadi Atallah, El-Barramia, and Wadi Sodmien, indicating more favorable physical properties that are consistent with ASTM C97^38^ specifications. Nevertheless, their UCS values remain lower than those reported for serpentinites from these localities and the limits specified by ASTM C170^39^ (Table 2).

**Table 2:** Comparison of physical and mechanical properties of serpentinite from Umm Khasila (the study area) and serpentinites from other Egyptian localities (Wadi Atallah, El-Barramia, and Wadi Sodmien^18^.

	Wadi Atallah	Wadi Sodmien	Barramia	Um Khasila	Test Methods	ASTM requirements
Bulk density (g/cm^3^)	2.60	2.60	-	2.72	C97	2.56 (min)
Water absorption (%)	0.23	0.23	0.20	0.10	C97	0.20max./0.60max.
Apparent porosity (%)	0.61	0.61	4.12	0.29	-	-
Uniaxial Compressive strength (MPa)	148.7	147.6	89	67.33	C170	69 (min)

**Fig. 8:**
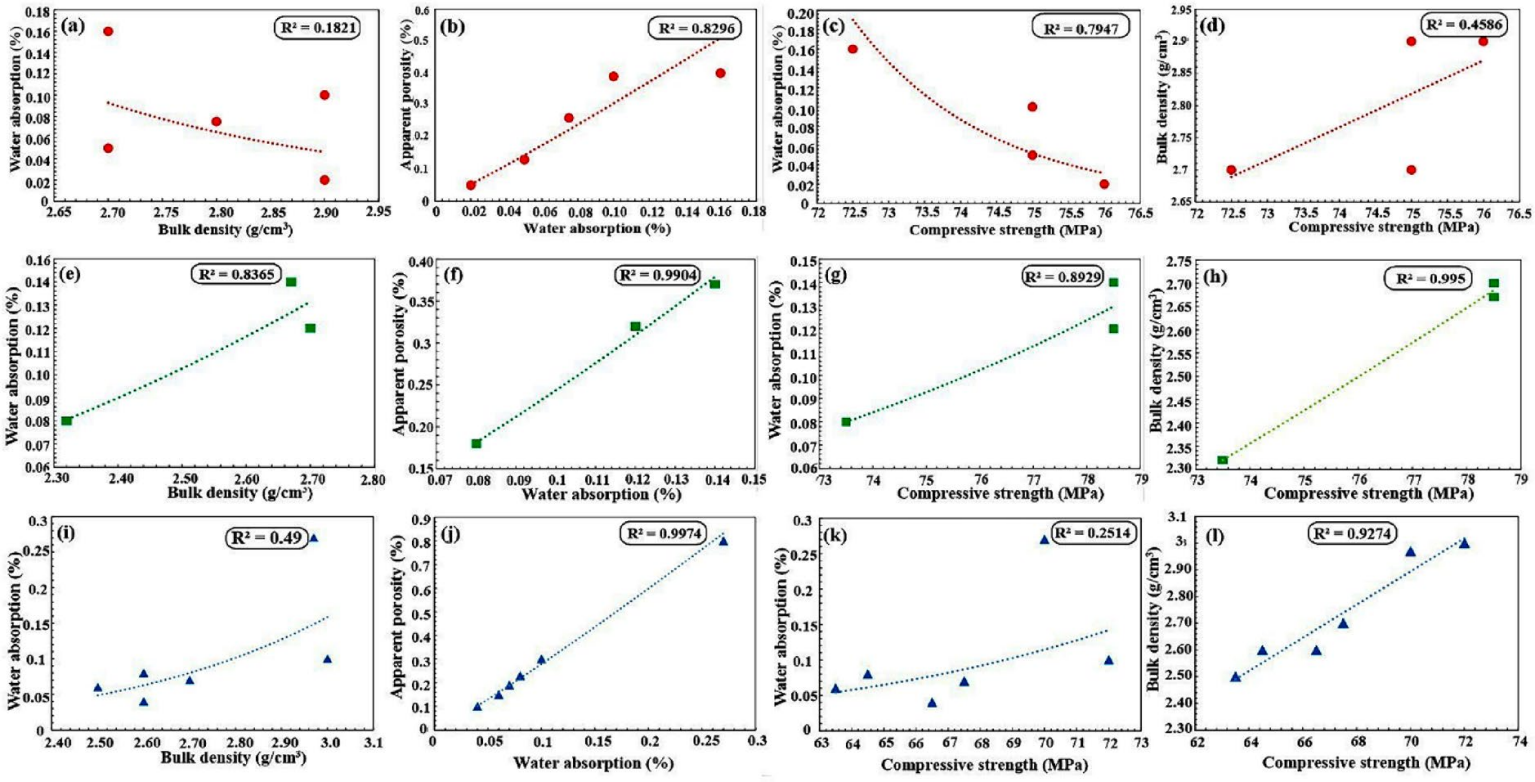
Binary plots illustrating the relationships between selected petrophysical properties for the metagabbro–diorite complex (**a–d**), olivine gabbro (**e–h**), and serpentinite (**i–l**). Each row represents one rock type and shows the relationships between bulk density (g/cm³) and water absorption (%) (**a**,** e**,** i**), water absorption (%) and apparent porosity (%) (**b**,** f**,** j**), compressive strength (MPa) and water absorption (%)(**c**,** g**,** k**), and compressive strength (MPa) and bulk density (g/cm³) (**d**,** h**,** l**).

Based on the combined physical and mechanical results, the suitability of the studied rock types for dimension-stone applications was evaluated in accordance with the relevant ASTM standards. For the metagabbro samples, the physico-mechanical relationships indicate consistent trends (Fig. 8a-d). Water absorption was included in the biplot analysis as it reflects pore connectivity and indirectly integrates the effects of porosity and alteration on mechanical behavior. Bulk density is inversely related to water absorption, while water absorption is positively related to apparent porosity. In addition, UCS decreases with increasing water absorption and increases with bulk density. Overall, these results suggest that metagabbros with higher bulk density and lower porosity tend to exhibit superior mechanical strength, indicating better potential for use as durable dimension stones.

For the olivine gabbro samples, the physico-mechanical relationships reveal some distinctive trends (Fig. 8e-h). The bulk density shows a positive correlation with water absorption, which is atypical for most rocks but may reflect the influence of secondary alteration products and microfractures that increase water absorption despite the higher density^52^. A positive correlation is observed between water absorption and apparent porosity. UCS correlates positively with both bulk density and water absorption. However, this interpretation is based on correlations derived from only three data points and should therefore be considered preliminary, representing a limitation of the present study. Consequently, Olivine gabbro exhibits relatively high UCS and density, supporting its potential suitability for dimension-stone use.

In contrast, the serpentinite samples show lower UCS but display clear property relationships (Fig. 8i–l). Bulk density is positively correlated with both water absorption and UCS. Water absorption is correlated positively with apparent porosity. In addition, UCS shows a positive correlation with water absorption, which is a counterintuitive but well-documented phenomenon. Unlike most rocks, where water absorption typically weakens the structure, serpentinites can exhibit an increase in strength with higher water content up to a point, directly linked to their unique mineralogy and the two distinct roles of water: chemically bound structural water and physically absorbed pore water. Also, serpentine minerals (antigorite, lizardite, chrysotile) are hydrous phyllosilicates^53^. Their strength derives from a layered structure in which a brucite [Mg(OH)₂] layer is fused to a silica tetrahedral layer. The hydroxyl (OH⁻) groups are an integral part of the crystal lattice. This structural water (up to ~ 13 wt%) is not a pore fluid but a fundamental building block^53,54^. The hydrogen bonding between these OH groups within and between the crystal layers provides significant cohesive strength. A well-crystallized, fully serpentinized rock has its framework “stitched together” by this hydrogen-bond network. Rocks with a higher degree of serpentinization (thus a higher bound-water content) often have a more complete, interlocking mesh texture of serpentine minerals, which can enhance mechanical integrity compared to a partially serpentinized, heterogeneous rock^54,55^.

It is hypothesised that, in serpentinites, the presence of veinlets represents an additional factor affecting UCS. Veinlets may act as mechanical discontinuities, and their influence on strength depends on the nature of the infilling minerals, vein thickness, and orientation relative to the loading direction. Veinlets filled with softer or altered minerals may locally reduce strength, whereas veins containing harder minerals do not necessarily enhance mechanical performance. Although veinlet characteristics were not quantitatively assessed in this study, they likely contribute to the observed variability in UCS and should be considered in future investigations. This interpretation is consistent with previous studies^56^, which emphasized the significant role of mineral veins and their orientation in controlling stone performance.

Overall, these observations suggest that, despite their acceptable durability, serpentinites generally exhibit lower UCS than gabbroic rocks, which may limit their suitability for use as dimension stones.

### Salt crystallization and thermal shock

**Fig. 9:**
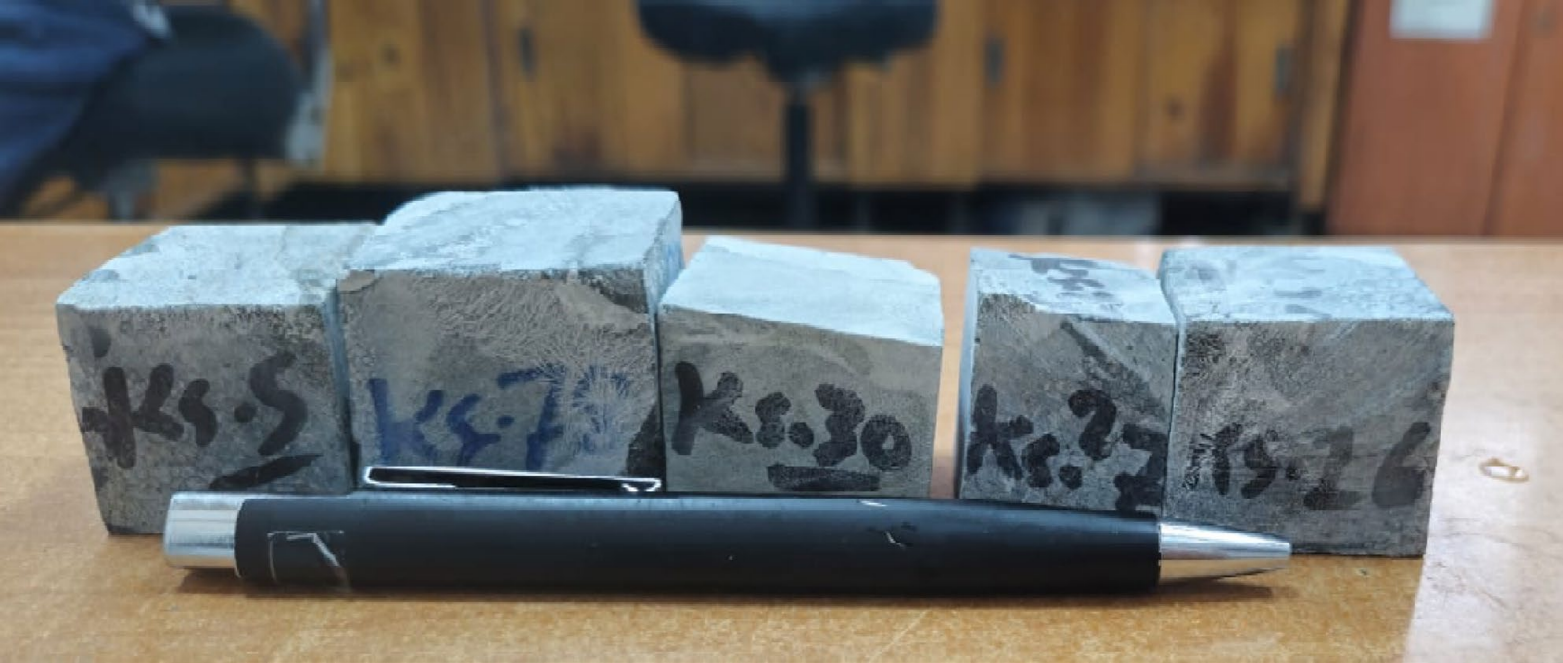
Formation of surficial salts after suffering 15 salt crystallization cycles.

Two durability tests were performed on serpentinite samples to evaluate their resistance to salt crystallization and thermal shock. The effects of these tests were assessed using two key parameters: the first is visual changes, also known as aesthetic changes, while the second is related to weight change. During the salt crystallization test, all serpentinite specimens exhibited visible efflorescence beginning from the fifth cycle, whereby the original dark green to olive coloration progressively faded to pale green, accompanied by the formation of friable surface salts (Fig. 9). On the other hand, after 50 thermal shock cycles, no noticeable aesthetic alterations such as cracking or shuttering were observed in the tested serpentinite samples.

**Fig. 10:**
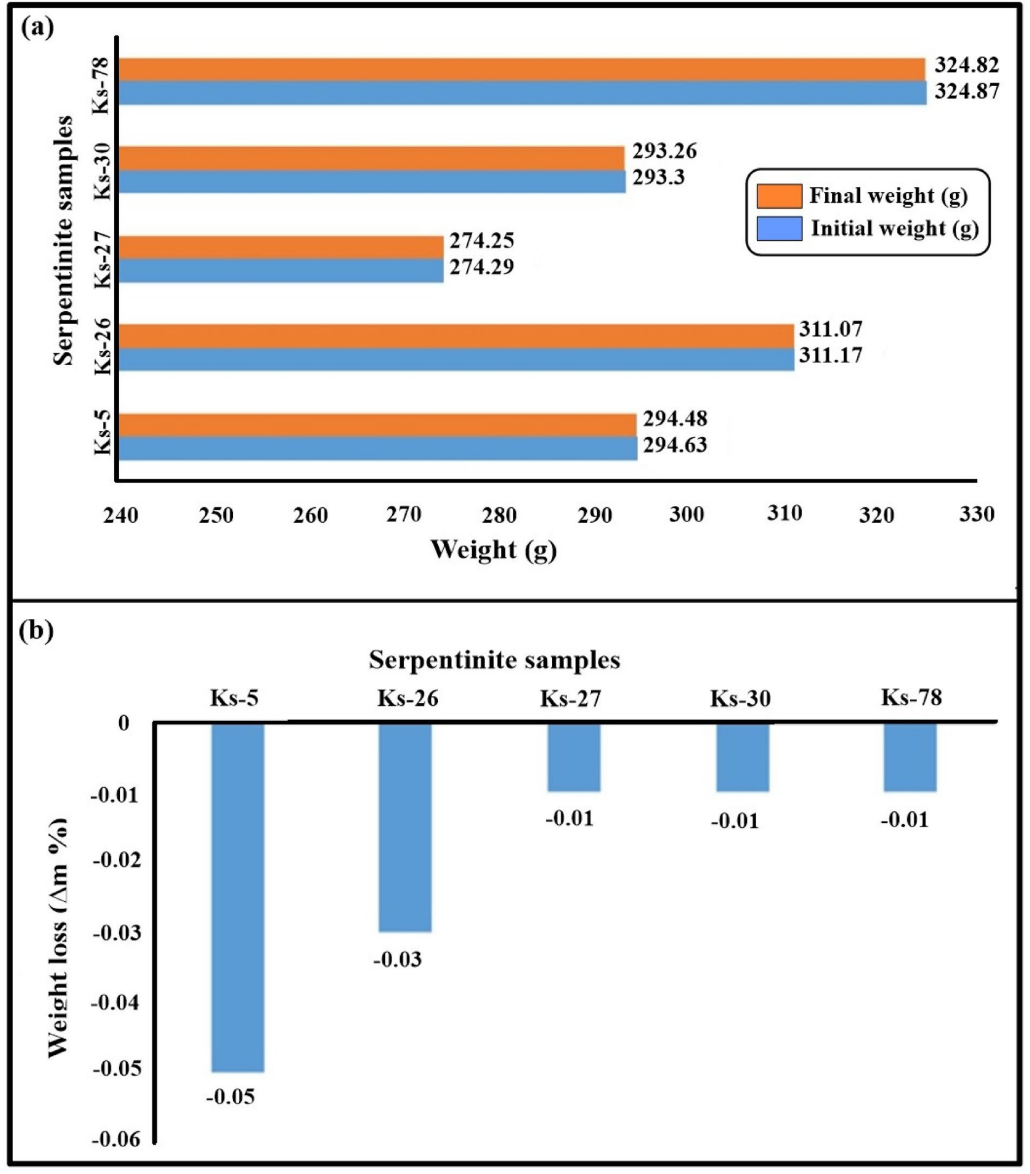
**(a)** Comparison between the initial and final weight of the serpentinite sample after 15 salt crystallization cycles. **(b)** The weight loss percentage % caused by 15 salt crystallization cycles.

**Fig. 11:**
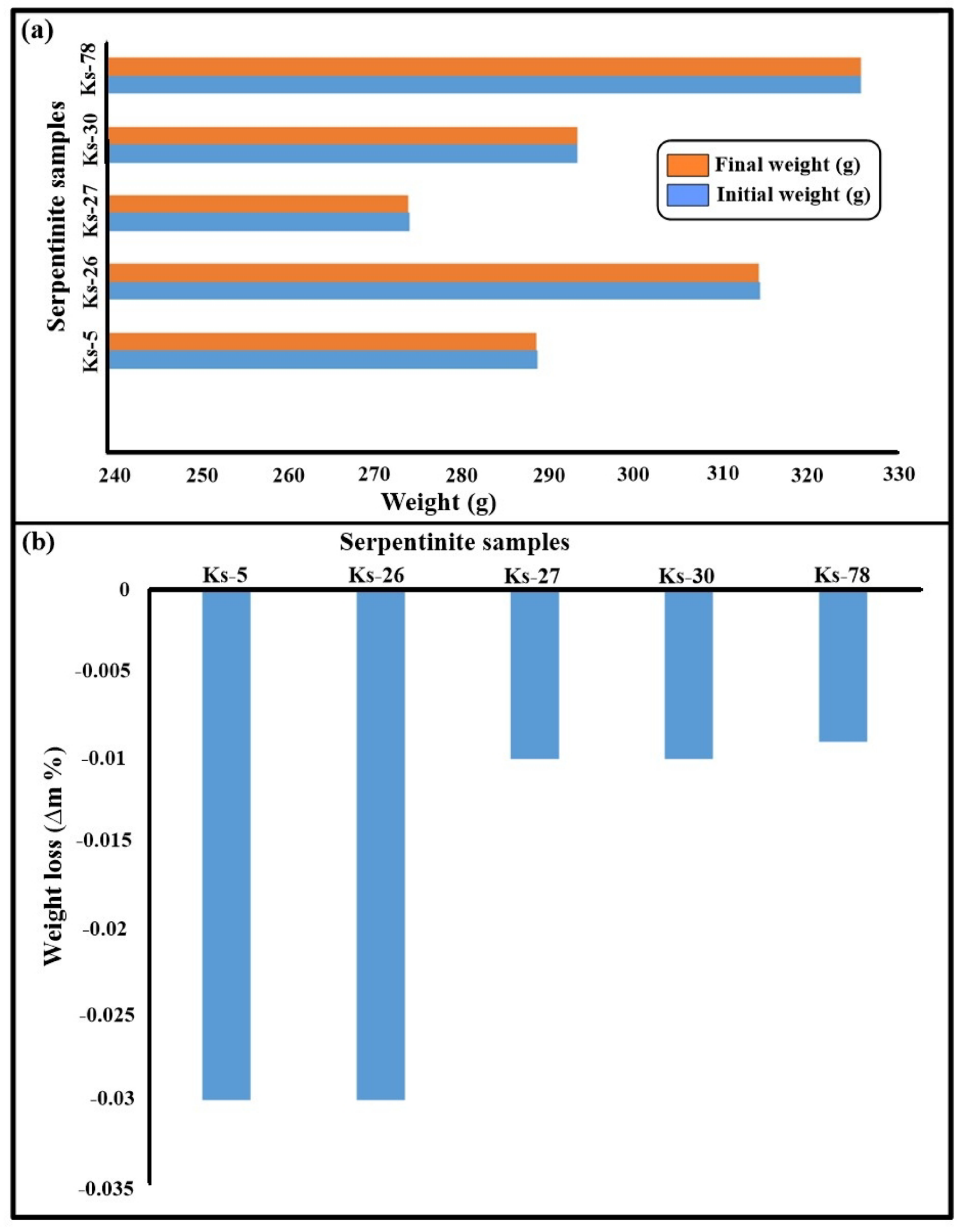
**(a)** Comparison between the initial and final weight of the serpentinite sample after 50 thermal shock cycles, **(b)** The weight loss percentage % caused by 50 thermal shock cycles.

Tables 3 and 4 summarize the weight changes of the serpentinite samples after 15 salt crystallization cycles and 50 thermal shock cycles, respectively. The results indicate that the serpentinite samples exhibited minor color changes and limited mass variation, with weight loss remaining below 1% throughout the applied cycles (Figs. 10 and 11). This behavior suggests a relatively stable response of the samples under the tested salt weathering and thermal shock conditions.

**Table 3:** The results of weight change throughout 15 salt crystallization cycles for serpentinite samples.

Sample code	Initial weight (g)	Final weight (g)	Weight change (+/- %)
Ks-5	294.63	294.48	−0.05
Ks-26	311.17	311.07	−0.03
Ks-27	274.29	274.25	−0.01
Ks-30	293.30	293.26	−0.01
Ks-78	324.87	324.82	−0.01

**Table 4:** The results of weight change throughout 50 thermal shock cycles for serpentinite samples.

Sample code	Initial weight (g)	Final weight (g)	Weight change (+/- %)
Ks-5	288.93	288.84	−0.03
Ks-26	314.27	314.17	−0.03
Ks-27	274.33	274.30	−0.01
Ks-30	293.50	293.46	−0.01
Ks-78	325.77	325.74	−0.009

### Relationship between UCS and petrophysical properties

The potential of rocks as dimension stone is mainly determined by their petrographic characteristics and physico-mechanical properties^1^. Petrographic analysis (Sect. 4.1) indicates that mineralogical composition and textural characteristics may contribute to the observed variations in UCS among the studied samples.

In the studied gabbroic rock, UCS values are relatively high (72.5–78.5 MPa), reflecting its mineralogical composition. These rocks are primarily composed of hard minerals like plagioclase (30–60%), amphibole (5–45%), quartz (< 5 vol%), olivine (5–20%), and pyroxene (2–5%), with the latter contributing to the highest strength values, Although pyroxene occurs in relatively minor amounts, it participates in an interconnected crystalline framework that may contribute to reinforcing the rock structure. Furthermore, the presence of amphiboles, which are characterized by relatively high hardness, including hornblende, tremolite, and actinolite, may contribute to increased mechanical competence of the rock. In contrast, serpentinites display lower UCS values, averaging 67.3 MPa, compared to the average value of 74.6–76.8 MPa for the gabbroic rocks. This reduction in strength is attributed to their mineralogical makeup: the platy and fibrous nature of serpentine minerals (antigorite and talc) introduces weakness planes, whereas the interlocking textures of plagioclase, pyroxene and olivine in the gabbros provide higher resistance to applied stress.

These findings suggest that variations in UCS may be related to mineral proportions and textures, with quartz- and feldspar-rich assemblages generally associated with higher strength in the studied samples, whereas serpentine-dominated assemblages appear comparatively weaker.

### Limitations of the study and future scope

1. The number of analyzed samples is relatively limited and unevenly distributed among lithologies, particularly for olivine gabbro. This constraint may affect the statistical robustness of the regression analyses and limits the generalization of the observed correlations.
2. The collected samples may not fully capture the spatial variability and heterogeneity of the rock units across the study area, especially considering variations in alteration intensity and structural deformation within the district.
3. The influence of foliation direction, vein orientation, and textural anisotropy on UCS was not explicitly tested. Given that mechanical performance in serpentinites and deformed gabbroic rocks can be strongly affected by structural fabric, this represents an important limitation.
4. The study focused primarily on uniaxial compressive strength (UCS) and durability tests (thermal shock and salt crystallization). In this context, UCS should be regarded as a preliminary indicator of mechanical behavior rather than a comprehensive mechanical characterization. Other relevant engineering tests, such as tensile strength (Brazilian test), flexural strength, abrasion resistance, and ultrasonic pulse velocity, were not conducted and may provide additional insight into stone performance.
5. Durability assessments were conducted under controlled laboratory conditions, which may not fully replicate long-term weathering processes, environmental fluctuations, and in-situ stress conditions.
6. Future research should therefore incorporate more extensive and spatially representative sampling, detailed microstructural analyses, and long-term field monitoring to better constrain the effects of weathering and alteration on rock durability and mechanical performance.

## Conclusion

This study evaluates the engineering properties of mafic–ultramafic rocks from the Atud–Um Khasila area to assess their suitability for building and dimension-stone applications. Mafic and ultramafic lithologies, particularly gabbro and serpentinite, are of economic importance owing to their durability and aesthetic appeal, making them potential candidates for dimension stone. The research aims to assess their suitability as construction materials through a detailed investigation of their physical and mechanical properties. A total of eighteen representative samples, comprising metagabbro, olivine gabbro, and serpentinite, were subjected to comprehensive physical and mechanical analyses. Standardized ASTM and EN procedures were applied to determine bulk density, water absorption, apparent porosity, and uniaxial compressive strength. Durability was further assessed in serpentinite samples through salt crystallization and thermal shock tests. The main findings are summarized as follows:

- 1-Petrographic examination identified key mineralogical constituents and textural features that contribute to variations in uniaxial compressive strength (UCS) of the studied rock.
- 2-The results show that metagabbro and olivine gabbro generally exhibit higher densities, lower porosities, and greater mechanical strength than serpentinite, likely due to their interlocking textures and mineral composition.
- 3-Serpentinite is characterized by relatively low apparent porosity and water absorption, indicating more favorable physical properties while exhibiting lower and more variable UCS values. This mechanical behavior appears to be influenced by its mineralogical composition, dominated by platy serpentine minerals, as well as by alteration processes. Despite the reduced strength, its durability, as evaluated through thermal shock and salt crystallization tests, remains within acceptable limits.
- 4-Based on the measured UCS values and within the limitations of the present dataset, most serpentinite samples may not meet ASTM requirements for dimension-stone applications. However, two samples (Td82 and Td92) showed relatively better performance, emphasizing the influence of mineralogy and alteration on the engineering behavior of these rocks.
- 5-Overall, the study highlights the potential influence of mineralogy and alteration processes in influencing the engineering performance of mafic–ultramafic rocks and their potential use as construction materials.

## Data Availability

The data supporting the findings of this study are available within the article (tables and figures).
